# Long-term survival of a patient with liver metastases from clear cell gastric adenocarcinoma after multimodality treatment including interventional oncology techniques: case report

**DOI:** 10.1186/s12876-022-02150-y

**Published:** 2022-03-07

**Authors:** Vesna Jugovec, Jernej Benedik, Jera Jeruc, Peter Popovic

**Affiliations:** 1 Department of Radiology, Institute of Oncology Ljubljana, Zaloška Cesta 2, 1000 Ljubljana, Slovenia; 2 Division of Medical Oncology, Institute of Oncology Ljubljana, Zaloška Cesta 2, 1000 Ljubljana, Slovenia; 3 Institute of Pathology, Faculty of Medicine, University of Ljubljana, Korytkova 2, 1000 Ljubljana, Slovenia; 4 Institute of Radiology, University Medical Centre Ljubljana, Zaloška Cesta 7, 1000 Ljubljana, Slovenia; 5 Department of Radiology, Faculty of Medicine, University of Ljubljana, Vrazov Trg 2, 1000 Ljubljana, Slovenia

**Keywords:** Gastric cancer, Hepatic metastases, Multidisciplinary approach, Interventional oncology, SIRT, Portal vein embolization, TACE, Liver resection

## Abstract

**Background:**

Gastric cancer (GC) is the fourth most common cancer and the third leading cancer-related cause of death worldwide since most patients are diagnosed at an advanced stage. The majority of GCs are adenocarcinomas (ACs), and the poorly characterized clear cell AC represents a unique subgroup of GCs and is an independent marker of poor prognosis. Even though the prognosis for patients with advanced GC is poor we present a report of a patient with long-term survival despite having liver metastases from clear cell gastric AC.

**Case presentation:**

A 45-year-old male with clear cell gastric AC underwent subtotal gastrectomy and postoperative chemoradiation. Only a year and a half after his initial treatment the disease spread to his liver. He received two lines of chemotherapy treatment within the next two years before a right hepatectomy was suggested. Due to an initially insufficient future liver remnant (FLR), transarterial chemoembolization (TACE) and portal vein embolization (PVE) were performed, which made the surgical procedure possible. Shortly after a disease progression in the remaining liver was detected. In the following three years the patient was treated with a carefully planned combination of systemic therapy and different interventional oncology techniques including selective internal radiation therapy (SIRT) and TACE. And as illustrated, an attentive, patient-tailored, multimodality treatment approach can sometimes greatly benefit our patients as he had an overall survival of 88 months despite the poor prognosis of his disease.

**Conclusion:**

To the best of our knowledge, this report is the first to describe a patient with liver metastases from clear cell gastric AC treated with interventional oncology techniques (PVE, TACE, and SIRT) in combination with other locoregional and systemic therapies thereby presenting that these interventional oncology techniques can be successfully integrated into long-term management of non-conventional liver tumors.

## Background

Gastric cancer (GC) is the fourth most common cancer and the third leading cancer-related cause of death worldwide because 80% to 90% of patients in countries without screening programs are diagnosed at an advanced stage [1]. 90% of all GCs are adenocarcinomas (AC), and the poorly characterized clear cell AC represents a unique subgroup of GCs and is an independent marker of poor prognosis [2].

Surgical treatment is potentially curative, especially at early stages. Nevertheless, most of the patients still develop liver metastases following resection; thus, combined modality therapies are standard for advanced stage disease [3].

Due to its rich, dual vascularization, the liver is an organ prone to metastases. Many solid cancers, including GC, metastasize to the liver, and very often the liver is the first and only site of metastatic disease.

The prognosis of patients with liver metastases is poor. If possible, hepatic resection is performed, but the vast majority of patients are unfortunately unresectable at the time of diagnosis of liver metastases. Chemotherapy regimens typically used in unresectable patients lead to a median overall survival (OS) of approximately 12 months [3].

When patients prove unresectable because of insufficient remnant liver volume, portal vein embolization (PVE) is one of the methods offered to stimulate growth of the future liver remnant (FLR), thereby sustaining the possibility of extensive liver resection.

Apart from chemotherapy and surgical resection, it is also possible to utilize local treatments such as transarterial chemoembolization (TACE), radiofrequency and microwave ablation, stereotactic body radiation therapy, and Yttrium-90 selective internal radiation therapy (Y-90 SIRT). TACE and SIRT are liver-directed therapies for primary and secondary liver malignancies with the aim of palliating and prolonging survival [4]. Several clinical studies have confirmed the benefits of drug-eluting beads loaded with doxorubicin chemoembolization (DEBDOX-TACE) and SIRT with respect to improved tumor response, reduced adverse events, and improved survival [5].

Recently, a combined analysis of three multicentric, randomized clinical trials has demonstrated an improvement in radiological response rate and hepatic progression-free survival in patients with metastatic colorectal cancer treated with SIRT [6]. SIRT and TACE have also been used to treat liver metastases from other primary tumors, including breast cancer, uveal melanoma, neuroendocrine tumors, and pancreatic cancer.

Publications of long-term outcomes in patients with clear cell gastric AC with liver metastases who have received SIRT or DEBDOX-TACE are currently lacking. Therefore, we present the first report of a patient with liver metastases from clear cell gastric AC treated with interventional oncology techniques in combination with surgery, radiotherapy, and systemic therapy (Table 1). We hope this case adds to the literature and expands treatment considerations in this difficult-to-manage patient group.

**Table 1:** Timeline table summary of the patient’s clinical course from December 2010 to May 2018 listing the main interventions and outcomes

Dates	Intervention	Outcome
December 2010	EGDS due to epigastric pain and persistent unproductive cough	A malignant lesion of the gastric antrum was discovered
February 2011	Subtotal gastrectomy and gastrojejunostomy	Clear cell gastric adenocarcinoma, R0 resection, stage IIb (pT4aN0M0)
March 2011–July 2011	Postoperative chemoradiotherapy	–
August 2012	Follow-up imaging	PD (two liver metastases)
October 2012–March 2013	First-line systemic therapy	PR
February 2014	Follow-up imaging	PD (enlargement of liver metastases)
March–May 2014	Second-line systemic therapy	Stopped after 3 cycles due to side effects
May 2014	Follow up imaging	SD, right hepatectomy suggested, but not feasible due to insufficient FLR (25%)
July–August 2014	TACE, PVE	SD, sufficient FLR achieved (41%)
October 2014	Right hepatectomy	R0 resection
January 2015	Follow up imaging	PD (two new liver metastases)
January–May 2015	Third-line systemic therapy	PD (enlargement of one of the liver metastases)
July 2015	SIRT	CR in target lesion
December 2015	Follow up imaging	PD (three new liver metastases)
December 2015	SIRT	CR
June 2016	Follow up imaging	PD (enlargement of liver metastases)
July 2016	SIRT	PR
December 2016	Follow up imaging	PD (enlargement of liver metastases)
February 2017	TACE	CR in target lesions
March–November 2017	Fourth-line systemic therapy	Disease progression (new liver metastases, ascites, and pleural effusions)
December 2017–May 2018	Hospice care	Death

*CR* complete response; *EGDS* esophagogastroduodenoscopy; *FLR* future liver remnant; *PD* progressive disease; *PR* partial response; *PVE* portal vein embolization; *SD* stable disease; *SIRT* selective internal radiation therapy with Y-90; *TACE* transcatheter arterial chemotherapy infusion

## Case report

In December 2010, a previously healthy 45-year-old male with no significant family medical record was presented with a history of epigastric pain accompanied by a persistent non-productive cough. A subsequent esophagogastroduodenoscopy identified a malignant lesion of the gastric antrum. Additional imaging examinations (abdominal ultrasound and chest X-ray) did not reveal any distant metastases. The patient's management at this point as well as during all its later stages was determined by a multidisciplinary tumor board (MTB).

In February 2011, the patient underwent an R0 subtotal gastrectomy followed by Billroth II gastrojejunostomy with a side-to-side jejuno-jejuneal (Braun) anastomosis.

**Fig. 1:**
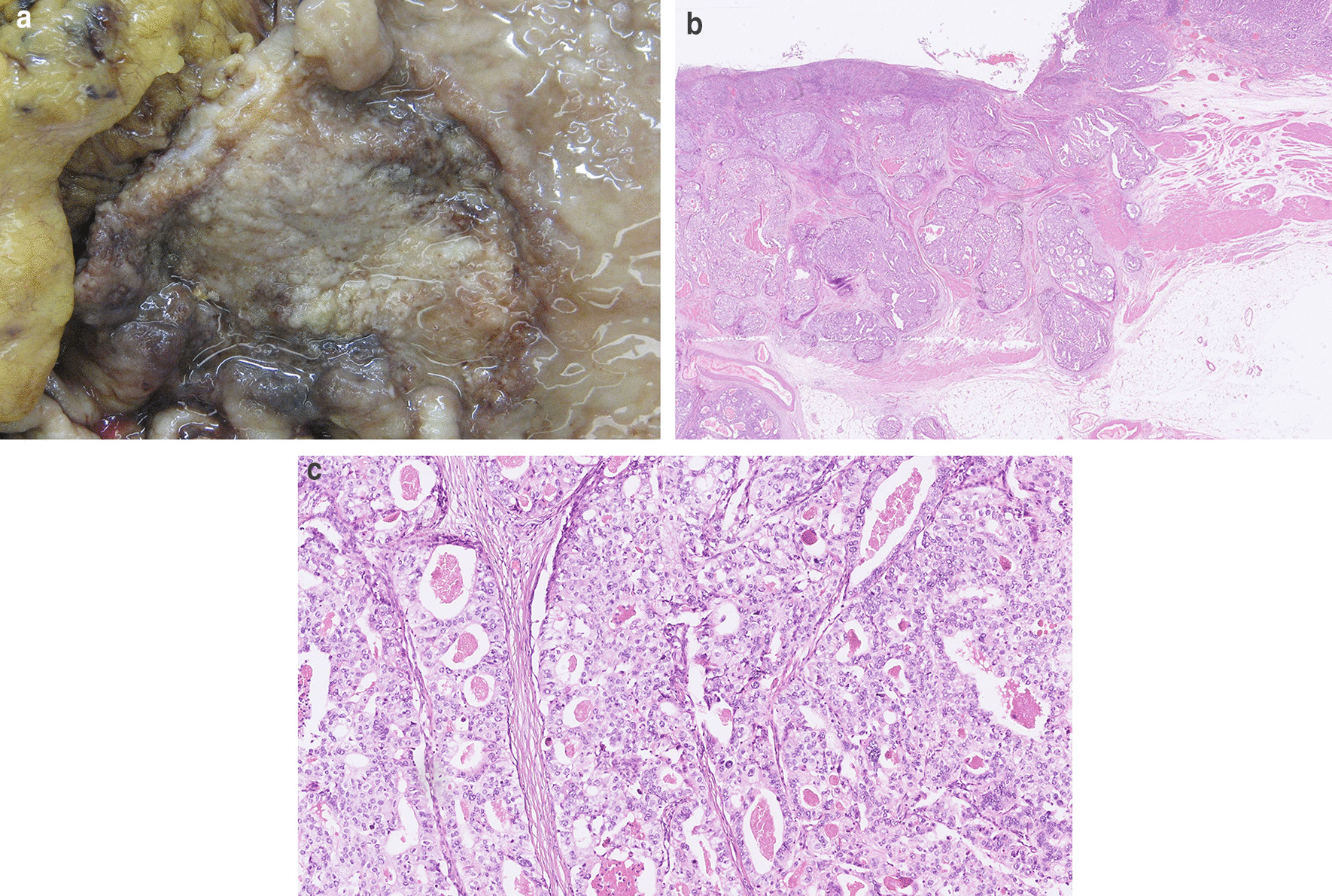
**A** A macroscopic examination of the gastrectomy specimen revealed a sharply demarcated and ulcerated tumour. **B** The histological examination revealed a clear cell adenocarcinoma with transmural infiltration of the gastric wall and extensive vascular invasion. **C** On higher magnification the tumour exhibited tubular and cribriform architecture

The subsequent histopathological examination revealed a sharply demarcated, exulcerated, moderately differentiated, clear cell intestinal-type invasive gastric AC (Fig. 1A–C). The 10 mm thick tumor, measuring 5 × 6 cm, infiltrated the serosal surface, and extensive lymphovascular invasion was also noted. The adjacent gastric mucosa showed signs of Helicobacter pylori gastritis with moderate intestinal metaplasia. All of the 18 dissected lymph nodes were negative for carcinoma. Based on this information and by taking into account the 7th edition of the American Joint Committee on Cancer TNM staging classification for gastric cancer, the described tumor fulfilled the criteria of a stage IIb carcinoma (pT4aN0M0).

From March to July 2011, the surgery was followed by postoperative chemoradiation with capecitabine, the total irradiation dose was 45 Gy delivered in 25 fractions over 5 weeks. In August 2012, two liver metastases with 5 cm and 1 cm diameters were discovered in the right liver lobe during follow-up imaging. From October 2012 until March 2013, first-line systemic chemotherapy was administered. The patient received 1 cycle of ECX (epirubicin, cisplatin, capecitabine); due to side effects, capecitabine was substituted with fluorouracil after the first cycle. A computed tomography (CT) scan after 3 cycles showed a partial response (PR) which was sustained on regular follow-ups post therapy.

**Fig. 2:**
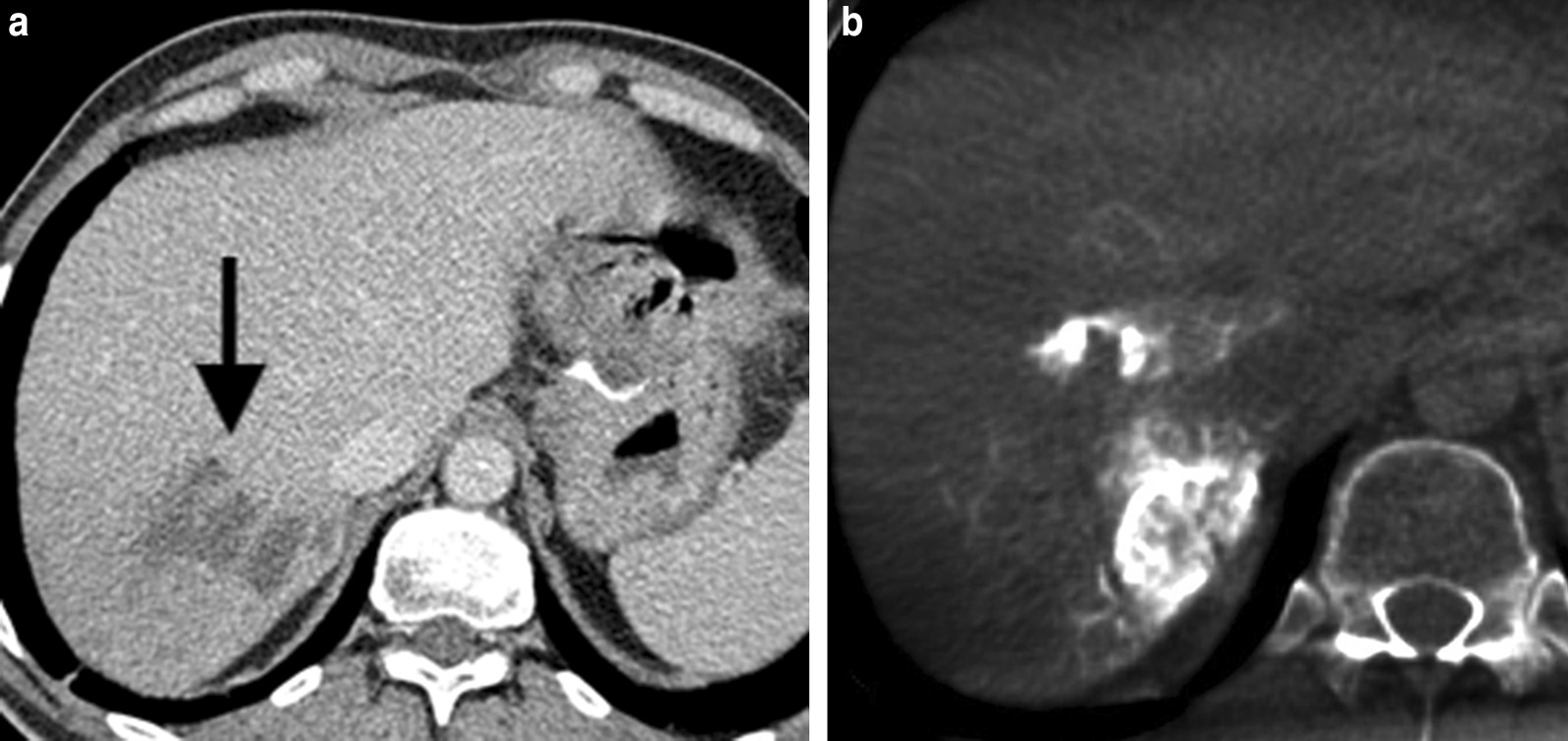
**A** Axial portal phase contrast-enhanced computed tomography image shows liver metastases in segment VII. **B** Cone-beam computed tomography prior to superselective drug-eluting beads loaded with doxorubicin transarterial chemoembolization (as a bridging therapy before the planned portal vein embolization) confirmed complete uptake of contrast media in the metastases

11 months after cessation of treatment, a CT scan identified enlargement of the existing liver lesions (progressive disease). From March until May 2014, the patient received 3 cycles of TOF regimen (paclitaxel, oxaliplatin, fluorouracil) as second-line systemic therapy. This chemotherapy was stopped due to side effects (febrile neutropenia and stomatitis). In May 2014, a surveillance CT scan revealed only an insignificant shrinkage of the liver metastases (stable disease), and the MTB suggested a right hepatectomy (Fig. 2A).

Due to a FLR of only 25%, an immediate resection was not feasible. Therefore, superselective DEBDOX-TACE was performed under the control of cone-beam computed tomography (*CBCT*) (Fig. 2B) in July and August 2014 with a 4-week interval as a bridging therapy before the planned portal vein embolization (PVE) which was performed one week after the last DEBDOX-TACE. Tandem 100 $\mathrm{\mu m}$ microspheres (Tandem®, Boston Scientific, Marlborough, MA, USA) loaded with 75 mg of doxorubicin were injected through a 2,4F microcatheter (Progreat®, Terumo Europe N.V, Belgium).

**Fig. 3:**
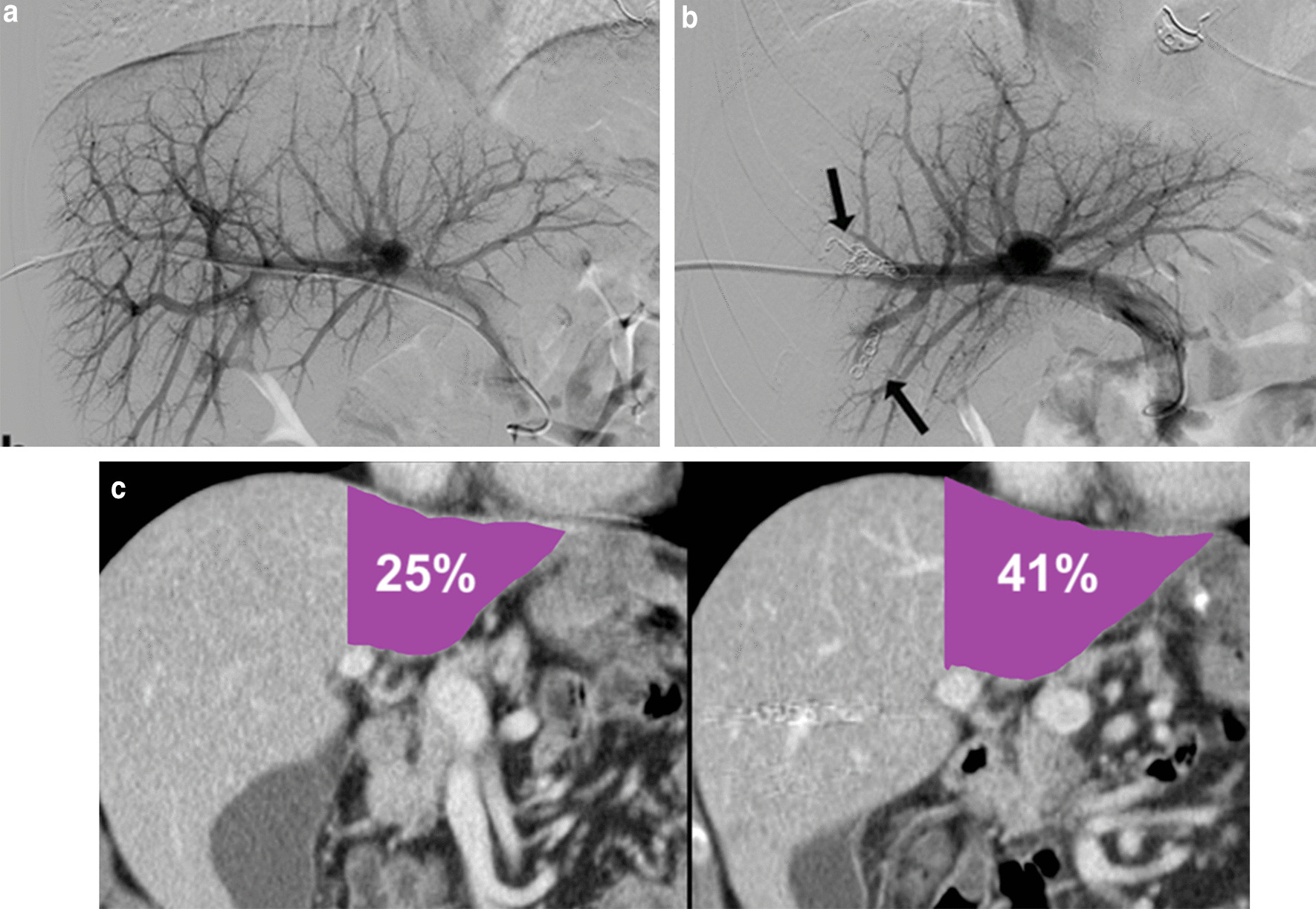
**A** Portography acquired immediately before portal vein embolization shows normal portal vein anatomy. **B** Control portography immediately after portal vein embolization with microspheres and coils shows successful occlusion of the right portal vein branches. **C** Computed tomography images before portal vein embolization and 4 weeks after showing significant hypertrophy of the left liver lobe (future liver remnant increased from 25 to 41%)

Transhepatic ipsilateral right PVE using 250–1000 $\mathrm{\mu m}$ particles (Embozene, Boston Scientific, Marlborough, MA, USA) and 6–10 mm embolization coils (Interlock-18 Fibered, Boston Scientific, Marlborough, MA, USA) was performed. Initially, smaller particles were infused to treat smaller distal branches followed by a stepwise increase in microsphere caliber size until near-complete stasis was achieved. Afterwards, embolization coils were placed centrally to prevent recanalization (Fig. 3A, B).

**Fig. 4:**
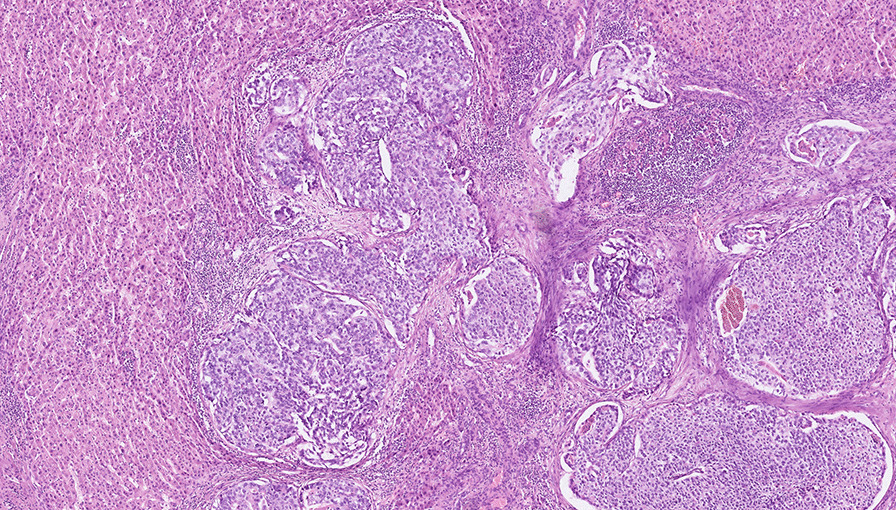
**A** Liver metastasis with similar histologic features as in the primary tumour

Total functional liver volume (TFLV) and FLR volume were measured before and 4 weeks after PVE to assess FLR, TFLV, and the FLR/TFLV ratio using CT imaging. In October 2014, the control CT scan after DEBDOX-TACE and PVE demonstrated stabilization of the ipsilateral disease burden and sufficient interval contralateral liver hypertrophy (with an FLR of 41%) which enabled a successful R0 right liver lobe resection (Fig. 3C). The histological examination of the resected liver metastases revealed similar histologic features as in the primary tumor (Fig. 4A).

**Fig. 5:**
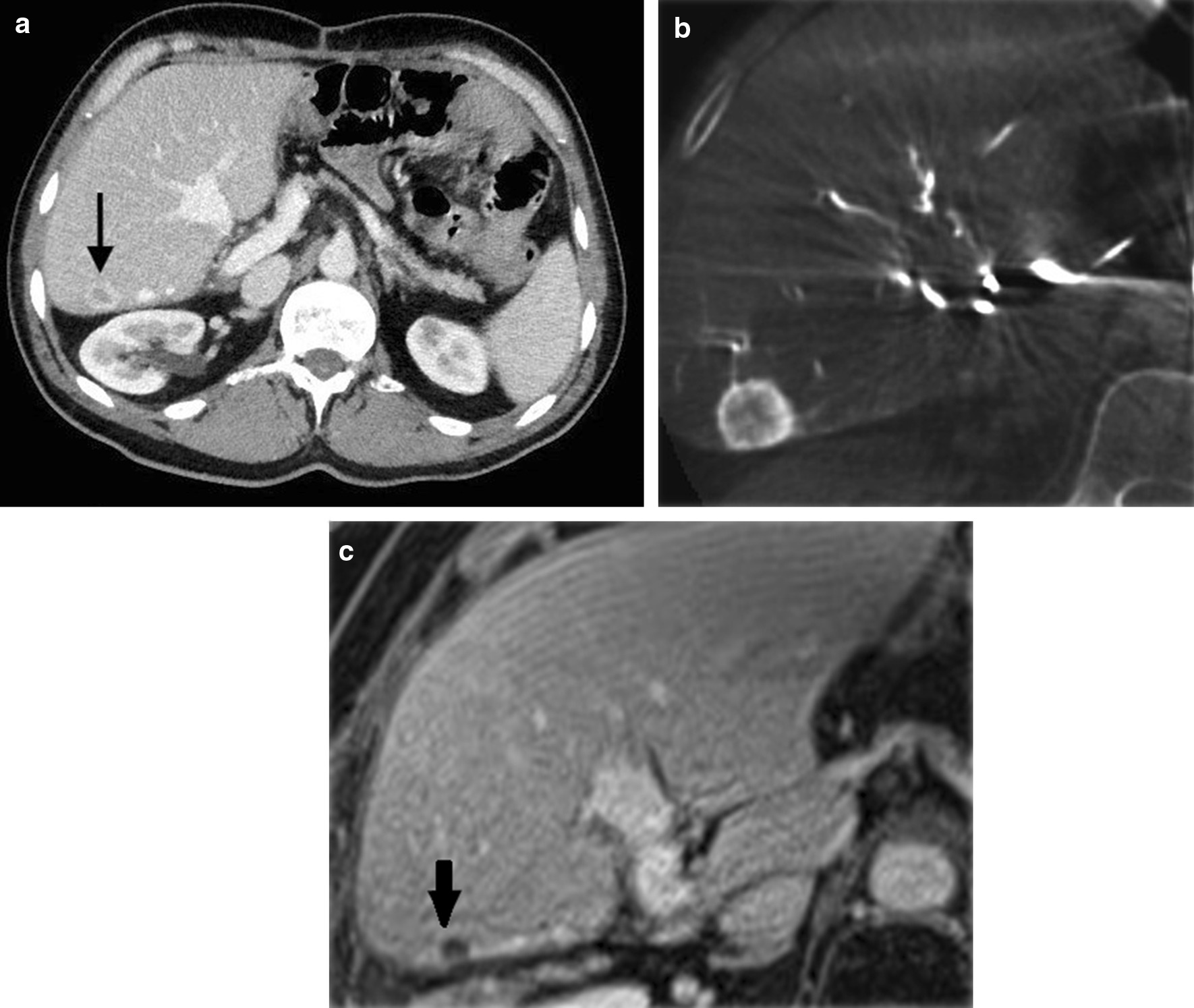
**A** Axial portal phase contrast-enhanced computed tomography image shows liver metastases in segment VI. **B** Cone-beam computed tomography prior to superselective Yttrium-90 selective internal radiation therapy confirmed complete uptake of contrast media in the metastases. **C** Follow-up magnetic resonance image shows complete response according to modified Response Evaluation Criteria in Solid Tumors

Following magnetic resonance imaging (MRI) in January 2015 which detected 2 small metastases in the remaining liver, the patient received 3 cycles of third-line systemic therapy containing FOLFIRI (folinic acid, fluorouracil, and irinotecan) between January and May 2015. The response evaluation CT revealed growth of one of the metastases (Fig. 5A); and in July 2015, superselective Y-90 SIRT (SIR‐Spheres®; Sirtex Medical, North Sydney, NSW, Australia) with an activity of 522 MBq was performed under CBCT control (Fig. 5B). Complete response in the target lesion was sustained (Fig. 5C). In December 2015, 3 new metastases were detected on control MR imaging. The patient was once again treated with superselective Y-90 SIRT (215 MBq + 241 MBq) under CBCT control, and a complete response lasting until June 2016 was achieved. In July 2016, the CT showed new metastases, and the last Y-90 SIRT (459 MBq) procedure was performed.

In December 2016, yet another liver disease progression was detected. This time despite the MTB's recommendation, another treatment with SIRT was not approved by the insurance company. Therefore, liver-directed therapy with DEBDOX-TACE was performed using Tandem 100 $\mathrm{\mu m}$ microspheres (Tandem®, Boston Scientific, Marlborough, MA, USA) loaded with 75 mg of doxorubicin in February 2017. In March 2017, the patient started receiving a fourth-line systemic therapy consisting of 3 cycles of Paclitaxel and Ramucirumab followed by a Ramucirumab maintenance therapy until a disease progression consisting of new liver metastases, ascites, and pleural effusions was noticed in November 2017.

In May 2018, 6 months after starting hospice care in December 2017, the patient died. His OS was 88 months, the last 69 months while having metastatic disease.

## Discussion/conclusion

Patients with GC usually present with advanced disease. Even the ones with early stages of the disease relapse locally or with distant metastases in 60% after curative resection [3]. The most common site for distant metastases is the liver, and liver lesions remain a major cause of death related to GC [3, 4].

If it is feasible, hepatic resection is offered to a highly selected group of patients, who are considering the number and size of metastases and the extent of liver involvement deemed resectable. But unfortunately, the vast majority of patients are unresectable at the time of diagnosis of liver metastasis. When patients prove unresectable because of insufficient remnant liver volume (less than 30% of standard liver volume in healthy liver or less than 40% in damaged liver parenchyma [7]), as was the case with our patient, PVE allows the possibility of extensive liver resection. The procedure induces hypertrophy of the non-diseased portion of the liver and thereby reduces the possibility of postoperative liver failure [7]. Liver cirrhosis has a negative effect on regeneration, but cholestasis and chemotherapy do not seem to have an influence on the hypertrophy response [7]. It has been shown that preoperative sequential selective TACE and PVE which can be performed to limit tumor growth and to increase the rate of FLR hypertrophy resulted in a high rate of complete tumor necrosis associated with longer recurrence-free survival [8]. Ogata et al. published an analysis of 36 patients with HCC and cirrhosis who underwent resection after PVE; 18 of these patients (50%) had TACE 3 weeks prior to PVE. The mean increase in FLR volume was higher for the TACE plus PVE group compared with PVE alone (12.5% versus 8.4%; *p* = 0.022). The TACE plus PVE group also had a significantly increased incidence of complete tumor necrosis (15 of 18 vs 1 of 18; *p* < 0.001) and a statistically significant higher 5-year disease-free survival (37% versus 19%; *p* = 0.041) [9]. To restrict tumor growth, the time between PVE and liver resection should be limited. In our case, sequential transarterial chemoembolization and PVE were successfully performed.

Palliative systemic treatment plays an important role in patients with inoperable and metastatic GC, and it has shown improved survival and quality of life compared with best supportive care alone. Doublet platinum/fluoropyrimidine combinations, as well as triplets containing anthracycline or taxans, are recommended for fit patients. As an alternative to platinum-based therapy, irinotecan plus leucovorin and infusional 5-FU (FOLFIRI) have been studied in both phase II trials and one phase III randomized trial in the first-line setting and may be considered for selected patients. Trastuzumab is recommended in conjunction with platinum and fluoropyrimidine-based chemotherapy for patients with HER2-positive advanced gastric cancer [3].

Over the last decade, the use of different intra-arterial therapies (IAT) has been reported as a palliative option in the treatment of liver metastases. IAT consist of bland embolization, chemotherapy-based modalities including conventional transcatheter arterial chemoembolization (cTACE), transcatheter arterial chemotherapy infusion (TACI), drug-eluting bead TACE, and intra-arterial brachytherapy-based modality—SIRT. One of the newest methods is SIRT which involves the administration of polymer microspheres 20–60 microns in size containing radioactive yttrium (90Y) directly into the tumor vasculature through a catheter and relies on the beta radiation emitted by the isotope to induce tumor necrosis and apoptosis [6, 10]. Observed side effects include abdominal pain, feeling of tightness in the abdomen, nausea, loss of appetite, and fever. Most often, these symptoms resolve within a week after the radioembolization [6, 10]. Our patient did not experience any of the described side effects at any time after being treated with SIRT on three different occasions.

Recent data shows that superselective SIRT and TACE under CBCT are associated with lower adverse events, a higher rate of tumor response, and increased survival [5, 10]. In superselective SIRT or TACE, proper identification of the tumor feeding arteries and detection of the target tumor is crucial. The use of SIRT and TACE has not been previously reported for patients with liver metastases from clear cell gastric adenocarcinoma.

Treatment response after DEBDOX-TACE and SIRT was evaluated with contrast enhanced CT or MR imaging with hepatospecific contrast media (Primovist®, Gd-GD-EOB-DTPA, Bayer Schering Pharma AG, Germany) 2 months after last DEBDOX-TACE and 3 months after SIRT according to mRECIST 1.1 criteria.

To the best of our knowledge, this report is the first to describe a patient with liver metastases from clear cell gastric adenocarcinoma treated with interventional oncology techniques in combination with other locoregional (surgery) and systemic therapies.

And as illustrated in our case report, an attentive, patient-tailored, multimodality treatment approach taking advantage also of different interventional oncology techniques at our disposal can sometimes greatly benefit our patients.

## Data Availability

Not applicable.

## Abbreviations

GC: Gastric cancer
AC: Adenocarcinoma
OS: Overall survival
PVE: Portal vein embolization
FLR: Future liver remnant
TACE: Transarterial chemoembolization
Y-90 SIRT: Yttrium-90 selective internal radiation therapy
SIRT: Selective internal radiation therapy (Y-90 SIRT)
DEBDOX-TACE: Drug-eluting beads loaded with doxorubicin transarterial chemoembolization
MTB: Multidisciplinary tumor board
CT: Computed tomography
PR: Partial response
CBCT: Cone-beam computed tomography
TFLV: Total functional liver volume
cTACE: Conventional transcatheter arterial chemoembolization
TACI: Transcatheter arterial chemotherapy infusion
